# Multiple analyses of large-scale genome-wide association study highlight new risk pathways in lumbar spine bone mineral density

**DOI:** 10.18632/oncotarget.8948

**Published:** 2016-04-23

**Authors:** Jinsong Wei, Ming Li, Feng Gao, Rong Zeng, Guiyou Liu, Keshen Li

**Affiliations:** ^1^ Department of Orthopedic Surgery, Affiliated Hospital of Guangdong Medical University, Zhanjiang, China; ^2^ Department of Endocrinology and Metabolism, The Fourth Affiliated Hospital of Harbin Medical University, Harbin, Heilongjiang, China; ^3^ Department of Trauma and Emergency Surgeon, The Second Affiliated Hospital, Harbin Medical University, Harbin, China; ^4^ Genome Analysis Laboratory, Tianjin Institute of Industrial Biotechnology, Chinese Academy of Sciences, China; ^5^ Institute of Neurology, Affiliated Hospital of Guangdong Medical University, Zhanjiang, China; ^6^ Stroke Center, Neurology & Neurosurgery Division, The Clinical Medicine Research Institute & The First Affiliated Hospital, Jinan University, Guangzhou, China

**Keywords:** osteoporosis, bone mineral density, pathway analysis, genome-wide association studies

## Abstract

Osteoporosis is a common human complex disease. It is mainly characterized by low bone mineral density (BMD) and low-trauma osteoporotic fractures (OF). Until now, a large proportion of heritability has yet to be explained. The existing large-scale genome-wide association studies (GWAS) provide strong support for the investigation of osteoporosis mechanisms using pathway analysis. Recent findings showed that different risk pathways may be involved in BMD in different tissues. Here, we conducted multiple pathway analyses of a large-scale lumbar spine BMD GWAS dataset (2,468,080 SNPs and 31,800 samples) using two published gene-based analysis software including ProxyGeneLD and the PLINK. Using BMD genes from ProxyGeneLD, we identified 51 significant KEGG pathways with adjusted *P*<0.01. Using BMD genes from PLINK, we identified 38 significant KEGG pathways with adjusted *P*<0.01. Interestingly, 33 pathways are shared in both methods. In summary, we not only identified the known risk pathway such as Wnt signaling, in which the top GWAS variants are significantly enriched, but also highlight some new risk pathways. Interestingly, evidence from further supports the involvement of these pathways in MBD.

## INTRODUCTION

Osteoporosis is a common human complex disease [1–2]. It is mainly characterized by low bone mineral density (BMD) and/or low-trauma osteoporotic fractures (OF), both of which have strong genetic determination [1–2]. However, the specific genes influencing these phenotypic are largely unknown [1–2]. Much effort has been put into identifying the genetic determinants of this disease, especially the genome-wide association studies (GWAS), which have recently provided rapid insights into genetics of osteoporosis [1–3].

In 2012, Estrada et al. performed the largest meta-analysis to date on lumbar spine BMD (LS-BMD; n = 31,800 cases) and femoral neck BMD (FN-BMD; n = 32,961), including 17 GWAS datasets from individuals of European and East Asian ancestry [3]. They replicated the top BMD-associated markers in 50,933 independent subjects and the risk of low-trauma fracture in 31,016 individuals with a history of fracture (cases) and 102,444 controls [3]. They identified 56 loci (32 new) associated with BMD at genome-wide significance [3]. In 2015, Zheng et al. reported novel non-coding genetic variants with large effects on BMD (n = 53,236) and fracture (n = 508,253) individuals of European ancestry from the general population, and identified EN1 as a determinant of bone density and fracture by whole-genome sequencing [4]. However, these newly identified susceptibility loci exert very small risk effects and cannot fully explain the underlying genetic risk. A large proportion of heritability has yet to be explained.

Pathway analyses of GWAS have been widely conducted in human complex diseases or phenotypes, such as Alzheimer's disease [5–8], rheumatoid arthritis [9–10] and body mass index [11]. The existing large-scale GWAS datasets provide strong support for the investigation of osteoporosis mechanisms using pathway analysis methods. Zhang et al. used a novel pathway-based analysis approach in wrist ultradistal radius BMD GWAS, examining approximately 500,000 single nucleotide polymorphisms (SNPs) from 984 unrelated whites [12]. They identified the regulation-of-autophagy pathway to be the most significant signal for association with wrist ultradistal radius BMD. They confirmed the regulation-of-autophagy pathway to be significantly associated with arm BMD in the Framingham Heart Study sample containing 2187 subjects [12].

Lee et al. performed a pathway analysis of hip BMD GWAS in 5,715 Europeans [13]. They identified eight significant pathways including gamma-hexachlorocyclohexane degradation, regulation of the smoothened signaling pathway, transmembrane activator and CAML-interactor and B cell maturation antigen stimulation of B cell immune response, endonuclease activity, regulation of defense response to virus, serine type endopeptidase inhibitor_activity, endoribonuclease activity, and myeloid leukocyte differentiation.

All these above findings show different risk pathways may be involved in BMD in different tissues. Here, we conducted multiple pathway analyses of a large-scale lumbar spine BMD GWAS using two published gene-based analysis methods including roxyGeneLD [14] and PLINK [15].

## RESULTS

### Pathway analysis of BMD genes from the ProxyGeneLD

Using ProxyGeneLD, these 2,468,080 SNPs are assigned to 16898 genes, which include at least one adjusted SNP. Using the 1236 significant genes (unadjusted *P*<0.05), we performed a pathway analysis. We identified 51 significant KEGG pathways (adjusted *P*<0.01). Based on the classifications of the KEGG pathways, these 51 pathways can be mainly divided into immune system and diseases (n=10), environmental information processing (n=10), cellular processes (n=8), cancers (n=6), and infectious diseases (n=3). These significant pathways were described in Table 1. All p-values of individual gene from ProxyGeneLD are described in Supplementary Table 1. The detailed gene information in significant KEGG pathways is described in Supplementary Table 2.

**Table 1:** Significant pathways with *P*<0.01 by pathway analysis of BMD genes using the “best SNP per gene” method

Classifications	Pathway ID	Pathway Name	C	O	E	R	rawP	adjP
Immune diseases	hsa05323	Rheumatoid arthritis	91	16	2.61	6.14	6.45E-09	3.50E-07
Environmental Information Processing	hsa04310	Wnt signaling pathway	150	20	4.3	4.66	1.26E-08	4.16E-07
Immune diseases	hsa04940	Type I diabetes mellitus	43	11	1.23	8.93	2.52E-08	6.24E-07
Environmental Information Processing	hsa04350	TGF-beta signaling pathway	84	14	2.41	5.82	1.14E-07	1.88E-06
Cardiovascular diseases	hsa05416	Viral myocarditis	70	12	2	5.99	6.63E-07	9.38E-06
Immune diseases	hsa05330	Allograft rejection	37	9	1.06	8.49	7.60E-07	9.41E-06
Endocrine system	hsa04916	Melanogenesis	101	14	2.89	4.84	1.17E-06	1.29E-05
Cellular Processes	hsa04510	Focal adhesion	200	20	5.73	3.49	1.46E-06	1.45E-05
Immune diseases	hsa05332	Graft-versus-host disease	41	9	1.17	7.67	1.93E-06	1.74E-05
Cancers: Specific types	hsa05217	Basal cell carcinoma	55	10	1.57	6.35	3.24E-06	2.47E-05
Infectious diseases: Bacterial	hsa05150	Staphylococcus aureus infection	55	10	1.57	6.35	3.24E-06	2.47E-05
Cellular Processes	hsa04114	Oocyte meiosis	112	14	3.21	4.37	4.10E-06	2.71E-05
Environmental Information Processing	hsa04340	Hedgehog signaling pathway	56	10	1.6	6.24	3.85E-06	2.71E-05
Infectious diseases: Parasitic	hsa05140	Leishmaniasis	72	11	2.06	5.34	6.21E-06	3.84E-05
Cardiovascular diseases	hsa05412	Arrhythmogenic right ventricular cardiomyopathy (ARVC)	74	11	2.12	5.19	8.15E-06	4.75E-05
Environmental Information Processing	hsa04060	Cytokine-cytokine receptor interaction	265	22	7.59	2.9	9.53E-06	5.18E-05
Cancers: Specific types	hsa05210	Colorectal cancer	62	10	1.78	5.63	9.94E-06	5.18E-05
Immune diseases	hsa05320	Autoimmune thyroid disease	52	9	1.49	6.04	1.53E-05	7.57E-05
Immune diseases	hsa05310	Asthma	30	7	0.86	8.15	1.77E-05	8.01E-05
Nervous system	hsa04722	Neurotrophin signaling pathway	127	14	3.64	3.85	1.78E-05	8.01E-05
Immune system	hsa04062	Chemokine signaling pathway	189	17	5.41	3.14	3.48E-05	1.00E-04
Infectious diseases: Parasitic	hsa05145	Toxoplasmosis	132	14	3.78	3.7	2.75E-05	1.00E-04
Immune system	hsa04672	Intestinal immune network for IgA production	48	8	1.37	5.82	6.03E-05	2.00E-04
Cellular Processes	hsa04520	Adherens junction	73	10	2.09	4.78	4.33E-05	2.00E-04
Circulatory system	hsa04270	Vascular smooth muscle contraction	116	12	3.32	3.61	1.00E-04	3.00E-04
Cardiovascular diseases	hsa05410	Hypertrophic cardiomyopathy (HCM)	83	10	2.38	4.21	1.00E-04	3.00E-04
Cellular Processes	hsa04530	Tight junction	132	13	3.78	3.44	1.00E-04	3.00E-04
Cellular Processes	hsa04145	Phagosome	153	14	4.38	3.2	1.00E-04	3.00E-04
Genetic Information Processing	hsa03040	Spliceosome	127	13	3.64	3.57	7.62E-05	3.00E-04
Cancers: Specific types	hsa05221	Acute myeloid leukemia	57	8	1.63	4.9	2.00E-04	6.00E-04
Cellular Processes	hsa04110	Cell cycle	124	12	3.55	3.38	2.00E-04	6.00E-04
Immune system	hsa04612	Antigen processing and presentation	76	9	2.18	4.14	3.00E-04	8.00E-04
Cardiovascular diseases	hsa05414	Dilated cardiomyopathy	90	10	2.58	3.88	3.00E-04	8.00E-04
Genetic Information Processing	hsa03013	RNA transport	151	13	4.32	3.01	4.00E-04	1.10E-03
Cellular Processes	hsa04810	Regulation of actin cytoskeleton	213	16	6.1	2.62	5.00E-04	1.30E-03
Environmental Information Processing	hsa04514	Cell adhesion molecules (CAMs)	133	12	3.81	3.15	5.00E-04	1.30E-03
Endocrine system	hsa04910	Insulin signaling pathway	138	12	3.95	3.04	6.00E-04	1.50E-03
Environmental Information Processing	hsa04512	ECM-receptor interaction	85	9	2.43	3.7	7.00E-04	1.60E-03
Cancers: Specific types	hsa05213	Endometrial cancer	52	7	1.49	4.7	7.00E-04	1.60E-03
Cellular Processes	hsa04144	Endocytosis	201	15	5.76	2.61	7.00E-04	1.60E-03
Environmental Information Processing	hsa04010	MAPK signaling pathway	268	18	7.67	2.35	8.00E-04	1.80E-03
Cancers: Specific types	hsa05215	Prostate cancer	89	9	2.55	3.53	1.00E-03	2.30E-03
Cancers: Specific types	hsa05220	Chronic myeloid leukemia	73	8	2.09	3.83	1.10E-03	2.40E-03
Development	hsa04380	Osteoclast differentiation	128	11	3.67	3	1.20E-03	2.60E-03
Genetic Information Processing	hsa03050	Proteasome	44	6	1.26	4.76	1.50E-03	3.20E-03
Environmental Information Processing	hsa04070	Phosphatidylinositol signaling system	78	8	2.23	3.58	1.80E-03	3.70E-03
Immune diseases	hsa05322	Systemic lupus erythematosus	136	11	3.89	2.82	1.90E-03	3.80E-03
Neurodegenerative diseases	hsa05016	Huntington's disease	183	13	5.24	2.48	2.50E-03	5.00E-03
Endocrine system	hsa04914	Progesterone-mediated oocyte maturation	86	8	2.46	3.25	3.30E-03	6.40E-03
Environmental Information Processing	hsa04150	mTOR signaling pathway	52	6	1.49	4.03	3.60E-03	6.90E-03
Environmental Information Processing	hsa04020	Calcium signaling pathway	177	12	5.07	2.37	5.20E-03	9.70E-03

C, the number of reference genes in the category; O, the number of genes in the gene set and also in the category; E, expected number in the category; R, the ratio of enrichment, rawP, the p value from hypergeometric test; adjP, the p value adjusted by the multiple test adjustment.

### Pathway analysis of BMD genes from the PLINK

Using PLINK, these 2,468,080 SNPs are assigned to 16543 genes, which include at least one SNP. Using the 824 significant genes (unadjusted *P*<0.05), we performed a pathway analysis and identified 38 significant KEGG pathways (adjusted *P*<0.01). These 38 pathways can be mainly divided into immune system and diseases (n=10), cellular processes (n=8), environmental information processing (n=7), genetic and environmental information processing (n=3), infectious diseases (n=4). These significant pathways were described in Table 2. All p-values of individual gene from PLINK are described in Supplementary Table 3. The detailed gene information in significant KEGG pathways is described in Supplementary Table 4.

**Table 2:** Significant pathways with *P*<0.01 by pathway analysis of BMD genes using the meta-analysis method

Classifications	Pathway ID	Pathway Name	C	O	E	R	rawP	adjP	Shared
Immune diseases	hsa05323	Rheumatoid arthritis	91	12	1.61	7.45	7.60E-08	2.43E-06	Y
Environmental Information Processing	hsa04060	Cytokine-cytokine receptor interaction	265	19	4.69	4.05	3.31E-07	5.30E-06	Y
Immune diseases	hsa04940	Type I diabetes mellitus	43	8	0.76	10.52	7.77E-07	9.95E-06	Y
Immune diseases	hsa05320	Autoimmune thyroid disease	52	8	0.92	8.7	3.50E-06	3.73E-05	Y
Environmental Information Processing	hsa04010	MAPK signaling pathway	268	17	4.74	3.59	7.08E-06	6.47E-05	Y
Cellular Processes	hsa04145	Phagosome	153	12	2.71	4.43	1.97E-05	1.00E-04	Y
Cellular Processes	hsa04510	Focal adhesion	200	14	3.54	3.96	1.50E-05	1.00E-04	Y
Environmental Information Processing	hsa04310	Wnt signaling pathway	150	12	2.65	4.52	1.61E-05	1.00E-04	Y
Cardiovascular diseases	hsa05416	Viral myocarditis	70	8	1.24	6.46	3.32E-05	2.00E-04	Y
Cellular Processes	hsa04810	Regulation of actin cytoskeleton	213	14	3.77	3.72	3.03E-05	2.00E-04	Y
Immune diseases	hsa05330	Allograft rejection	37	6	0.65	9.17	4.38E-05	2.00E-04	Y
Infectious diseases: Parasitic	hsa05140	Leishmaniasis	72	8	1.27	6.28	4.08E-05	2.00E-04	Y
Immune diseases	hsa05332	Graft-versus-host disease	41	6	0.73	8.27	7.98E-05	3.00E-04	Y
Infectious diseases: Parasitic	hsa05145	Toxoplasmosis	132	10	2.34	4.28	1.00E-04	4.00E-04	Y
Immune diseases	hsa05310	Asthma	30	5	0.53	9.42	2.00E-04	6.00E-04	Y
Immune system	hsa04672	Intestinal immune network for IgA production	48	6	0.85	7.07	2.00E-04	6.00E-04	Y
Immune system	hsa04062	Chemokine signaling pathway	189	12	3.34	3.59	2.00E-04	6.00E-04	Y
Metabolism	hsa00250	Alanine, aspartate and glutamate metabolism	32	5	0.57	8.83	2.00E-04	6.00E-04	N
Cellular Processes	hsa04142	Lysosome	121	9	2.14	4.2	3.00E-04	9.00E-04	N
Immune system	hsa04612	Antigen processing and presentation	76	7	1.34	5.21	4.00E-04	1.10E-03	Y
Infectious diseases: Bacterial	hsa05150	Staphylococcus aureus infection	55	6	0.97	6.17	4.00E-04	1.10E-03	Y
Nervous system	hsa04722	Neurotrophin signaling pathway	127	9	2.25	4.01	4.00E-04	1.10E-03	Y
Environmental Information Processing	hsa04514	Cell adhesion molecules (CAMs)	133	9	2.35	3.82	6.00E-04	1.50E-03	Y
Environmental Information Processing	hsa04350	TGF-beta signaling pathway	84	7	1.49	4.71	7.00E-04	1.70E-03	Y
Environmental Information Processing	hsa04512	ECM-receptor interaction	85	7	1.5	4.65	8.00E-04	1.90E-03	Y
Cellular Processes	hsa04144	Endocytosis	201	11	3.56	3.09	1.00E-03	2.20E-03	Y
Genetic Information Processing	hsa03050	Proteasome	44	5	0.78	6.42	1.00E-03	2.20E-03	Y
Immune system	hsa04670	Leukocyte transendothelial migration	116	8	2.05	3.9	1.10E-03	2.30E-03	N
Cancers: Specific types	hsa05220	Chronic myeloid leukemia	73	6	1.29	4.65	1.90E-03	3.80E-03	Y
Cellular Processes	hsa04520	Adherens junction	73	6	1.29	4.65	1.90E-03	3.80E-03	Y
Development	hsa04380	Osteoclast differentiation	128	8	2.26	3.53	2.10E-03	4.10E-03	Y
Cellular Processes	hsa04530	Tight junction	132	8	2.34	3.43	2.50E-03	4.70E-03	Y
Infectious diseases: Viral	hsa05160	Hepatitis C	134	8	2.37	3.37	2.70E-03	4.90E-03	N
Cancers: Specific types	hsa05217	Basal cell carcinoma	55	5	0.97	5.14	2.90E-03	5.20E-03	Y
Environmental Information Processing	hsa04340	Hedgehog signaling pathway	56	5	0.99	5.05	3.10E-03	5.40E-03	Y
Cellular Processes	hsa04114	Oocyte meiosis	112	7	1.98	3.53	3.90E-03	6.60E-03	Y
Genetic Information Processing	hsa03013	RNA transport	151	8	2.67	2.99	5.60E-03	9.00E-03	Y
Genetic Information Processing	hsa03010	Ribosome	92	6	1.63	3.69	5.90E-03	9.20E-03	N

C, the number of reference genes in the category; O, the number of genes in the gene set and also in the category; E, expected number in the category; R, the ratio of enrichment, rawP, the p value from hypergeometric test; adjP, the p value adjusted by the multiple test adjustment. Y, this pathway is shared in pathway analysis of BMD genes using the “best SNP per gene” method; N, this pathway is not shared in pathway analysis of BMD genes using the “best SNP per gene” method;

### Shared KEGG pathways using both ProxyGeneLD and PLINK

We identified 33 pathways shared in pathway analyses of BMD genes using the “best SNP per gene” method and the meta-analysis method. These 33 pathways are related with immune system and diseases (n=9), cellular processes (n=7), environmental information processing (n=7), infectious diseases (n=3), genetic and environmental information processing (n=2). These shared pathways are highlighted in Table 2.

## DISCUSSION

Osteoporosis is a major public health problem. However, the specific genes or pathways influencing these phenotypic are largely unknown. Recently, two pathway analyses of MBD GWAS datasets have been conducted in wrist ultradistal radius and hip. Zhang et al. highlighted the regulation-of-autophagy pathway in wrist ultradistal radius BMD GWAS dataset [12]. Lee et al. identified eight significant pathways in hip BMD GWAS [13]. However, no shared pathway was identified in both studies. We think that different risk pathways may be involved in BMD in different tissues. Here, we conducted multiple pathway analyses of a large-scale lumbar spine BMD GWAS using the BMD genes from ProxyGeneLD [14] and PLINK [15]. Using BMD genes from ProxyGeneLD, we identified 51 significant KEGG pathways. Using BMD genes from PLINK, we identified 38 significant KEGG pathways. Interestingly, 33 pathways are shared in both methods.

We further searched the PubMed and Google Scholar databases to verify our findings. Interestingly, evidence further supports the involvement of these pathways in MBD. Take the Wnt signaling (hsa04310) for example. We identified it to be the most significant pathway (*P* = 3.26E-09) and 7^th^ significant pathway (*P* = 1.00E-04) using genes from the “best SNP per gene” method and the meta-analysis method, respectively. It is reported that Wnt signaling plays major roles in almost all aspects of skeletal development and homeostasis. Promising effective therapeutic agents for bone diseases are being developed by targeting the Wnt signaling pathway [16]. Wnt signaling regulates BMD through the lipoprotein receptor-related protein 5 (LRP5), a receptor of this signaling. Genetic variations at the LRP5 gene locus are associated with osteoporosis, which suggests that genetic variations in Wnt signaling genes may affect the pathogenesis of osteoporosis [17].

We further compared our results with previous GWAS [3, 18]. In 2008, Styrkarsdottir et al. also reported the involvement of RANK-RANKL-OPG pathway in BMD [18]. In 2012, Estrada et al. identified 56 loci associated with BMD at genome-wide significance (*P* < 5.00E-08) [3]. They applied the Gene Relationships Across Implicated Loci (GRAIL) text-mining algorithm to investigate connections between genes in 55 autosomal BMD-associated loci, and revealed significant (*P* < 0.01) connections between genes in 18 loci in three key biological pathways: the RANK-RANKL-OPG pathway (TNFRSF11A, TNFSF11 and TNFRSF11B); mesenchymal stem cell differentiation (RUNX2, SP7 and SOX9); and Wnt signaling (LRP5, CTNNB1, SFRP4, WNT3, WNT4, WNT5B, WNT16 and AXIN1) [3].

In addition to the Wnt signaling, there is also some literature evidence supporting the involvement of other risk pathways in BMD. More detailed information is described in Table 3.

**Table 3:** Literature evidences supporting that genes in measles pathway are associated with bone mineral density or osteoporosis

Pathway	Supporting evidence	Ref
Rheumatoid arthritis	BMD data of patients with low to moderately active RA demonstrated an association between high radiological RA damage and low BMD at the hip, which suggests an association between the severity of RA and the risk of generalised bone loss, which also occurred in corticosteroid naive patients [27]. There is a significant negative relationship between femoral neck BMD and disease duration in RA. The value of OR increases proportionately with lengthening of disease duration which can be reduced significantly by methotrexate therapy [28].	[27–28]
TGF-beta signaling pathway	TGF-beta is the possible Link between loss of bone mineral density and chronic inflammation [29]. TGF-β/BMPs have widely recognized roles in bone formation during mammalian development and exhibit versatile regulatory functions in the body [30].	[29–30]
Focal adhesion	Proline-rich tyrosine kinase 2 (PYK2), a member of the focal adhesion kinase family, plays a key role in the regulation of bone formation, and so inhibitors of this kinase might represent potential bone-building therapies for osteoporotic disease [31]. The focal adhesion, the actin cytoskeleton and cell-cycle are connected pathways and their genes are implicated in the pathogenesis of low BMD [32].	[31–32]
Type I diabetes mellitus	The lower BMD in type 1 versus type 2 diabetic patients and control subjects probably results from more rapid bone loss after the onset of type 1 diabetes [33]. patients with type 1 diabetes have a 6.9-fold increased incidence of hip fracture compared to controls [34].	[33–34]
Regulation of actin cytoskeleton	The focal adhesion, the actin cytoskeleton and cell-cycle are connected pathways [32]. Genes in these three pathways are implicated in the pathogenesis of low BMD [32]. Genome-wide linkage studies have highlighted the chromosomal region 3p14-p22 as a quantitative trait locus for BMD [35]. The *FLNB* gene, which is thought to have a role in cytoskeletal actin dynamics, is located within this chromosomal region and presents as a strong candidate for BMD regulation [35]. Mullin et al. identified significant associations between SNPs in the *FLNB* gene and BMD in Caucasian women [35].	[32, 35]

Until now, there are kinds of software tools for pathway analysis of the GWAS dataset [19]. Some tools including SNP ratio test [20], GenGen [21], GRASS [22], accept raw genotype datasets as input data. Other tools including ProxyGeneLD [14], ALIGATOR [19], i-GSEA4GWAS [19], and PLINK set-test [23], MAGENTA [24], and GESBAP [19] accept the summary results to subsequent pathway analysis. Here, we selected ProxyGeneLD and PLINK for gene-based test, as we did not have access to raw BMD genotype data. Both the ProxyGeneLD and PLINK have different approaches, assumptions regarding genomic architecture of risk variants in pathways, and different methods for the correction of LD and gene size effects. ProxyGeneLD produces a gene-wide p-value using the lowest p-value of the SNPs (the best SNP), or the lowest p-value in a cluster with multiple SNPs and clusters that fall within the gene boundaries [25]. The *P* value was adjusted for the LD patterns in the human genome and gene length. PLINK SET SCREEN TEST is a meta-analysis method that combines *P* values across all the SNPs in genes and adjusts for the LD [15].

Based on these different software tools for pathway analysis, we recognize some limitations using ProxyGeneLD and PLINK. First, the multiple testing corrections may not be sufficient to account for all biases in pathway analysis. The results from the BMD GWAS should be adjusted using a permutation test. However, the original SNP genotype data for each individual are not available to us now. When we get the SNP genotype data, we will further perform a pathway analysis using some available software such as SNP ratio test [20], GenGen [21], and GRASS [22]. These pathway analysis methods or software can be used to analyze the SNP genotype data, and can conduct a permutation test. Future replication studies using genotype data are required to replicate our findings.

In summary, we not only identified the known risk pathway such as Wnt signaling, in which the top GWAS SNPs are significantly enriched, but also highlight some new risk pathways. Interestingly, evidence from further supports the involvement of these pathways in MBD. We believe that our results advance our understanding of BMD mechanisms and will be very informative for future genetic studies in BMD. Further functional evaluation of this pathway to determine the mechanism by which it regulates BMD should be pursued to provide new insights into the pathogenesis of osteoporosis.

## MATERIALS AND METHODS

### The BMD GWAS dataset

The second lumbar spine BMD GWAS dataset used here comes from the summary results of a large-scale BMD GWAS conducted by Estrada et al [3], which is part of the GEnetic Factors for OSteoporosis consortium (GEFOS). Estrada et al. performed a meta-analysis of GWAS for BMD of the lumbar spine (LS-BMD; n=31,800) including ~2.5 million autosomal genotyped or imputed SNPs from 17 GWAS datasets from North America, Europe, East Asia and Australia [3]. BMD of the lumbar spine (LS-BMD) was measured in all cohorts using dual-energy X-ray absorptiometry following standard manufacturer protocols [3]. GWAS genotyping was followed by imputation to ~2.5 million SNPs from HapMap37 Phase II release 22 using Genome Build 36 [3]. Association between each SNP and BMD in each study was analyzed using sex-specific, age- weight- and principal components-adjusted standardized residuals under an additive genetic model [3]. In the end, we got the association results about 2,468,080 SNPs and BMD [3]. More detailed information is described in the original study [3].

### Gene-based testing for GWAS dataset by ProxyGeneLD

ProxyGeneLD assigns SNPs to specific genes [14]. This software flexibly takes into consideration the complex linkage disequilibrium (LD) patterns in the human genome and corrects for the inflation of significance caused by gene length. The LD information comes from the HapMap phase II CEU samples (release 22) [14]. Using the lowest p-value of the SNPs (the best SNP), or the lowest p-value in a cluster with multiple SNPs and clusters that fall within the gene boundaries, ProxyGeneLD produces a gene-wide p-value [25]. Intergenic SNPs, which is in high LD with the mapped genes, will have been mapped to genes for the next analysis.

### Gene-based testing for GWAS dataset by PLINK

PLINK is used to test for the GWAS dataset by a meta-analysis of all the SNPs in genes [15]. The set screen test uses an approximate Fisher's test to combine *P* values across all the SNPs in genes and adjusts for LD [15]. It is reported that Fisher's method is asymptotically optimal to get the overall significance by combining a set of *P*-values obtained from independent tests of the same null hypothesis (each SNP is not associated with disease) [15]. We applied this method to the BMD GWAS dataset using the LD information from the HapMap CEU population.

### Pathway-based testing for BMD GWAS dataset

The KEGG pathways in WebGestalt were used for pathway analysis (June 16, 2015) [26]. For a given pathway, a hypergeometric test was used to detect the overrepresentation of BMD-related genes among all of the genes in the pathway [26]. The *p*-value of *K* BMD-related genes in the pathway was calculated using

$$P=1-\sum_{i=0}^{K}\frac{C_{S}^{i}C_{N-S}^{m-i}}{C_{N}^{m}}$$

where *N* is the total number of genes of interest, *S* is the number of all of the BMD-related genes, *m* is the number of genes in the pathway and *K* is the number of BMD-related genes in the pathway.

In order to avoid testing overly narrow or broad pathways, we select WebGestalt KEGG pathways that contain at least 20 and at most 300 genes for subsequent analysis. The reference gene list is the entire entrez gene set. The minimum number of genes for a category is 5. The false discovery rate (FDR) method was used to correct for multiple testing. KEGG pathways with an adjusted *P*<0.01 are considered to be significantly associated with BMD.

## SUPPLEMENTARY TABLES










