# Supplementary material for: Multiple analyses of large-scale genome-wide association study highlight new risk pathways in lumbar spine bone mineral density
Source: Oncotarget. 2016 Apr 23;7(21):31429–39. doi: 10.18632/oncotarget.8948 (PMC5058768; doi:10.18632/oncotarget.8948)
Supplement: Supplementary file 1 [file oncotarget-07-31429-s001.pdf]

SUPPLEMENTARY TABLES

Supplementary Table S1: All p-values of individual gene from ProxyGeneLD

See Supplementary File 1

Supplementary Table S2: The detailed genes in significant KEGG pathways using gene from ProxyGeneLD

See Supplementary File 2

Supplementary Table S3: All p-values of individual gene from PLINK

See Supplementary File 3

Supplementary Table S4: The detailed genes in significant KEGG pathways using gene from PLINK

See Supplementary File 4
