# Supplementary material for: Multiple analyses of large-scale genome-wide association study highlight new risk pathways in lumbar spine bone mineral density
Source: Oncotarget. 2016 Apr 23;7(21):31429–39. doi: 10.18632/oncotarget.8948 (PMC5058768; doi:10.18632/oncotarget.8948)
Supplement: Supplementary file 3 [file oncotarget-07-31429-s003.doc]

**Supplementary Table 2**, The detailed genes in significant KEGG pathways using gene from ProxyGeneLD

| Pathway ID | Pathway Name | Gene |
| --- | --- | --- |
| hsa05323 | Rheumatoid arthritis | 3458 3122 3117 942 4050 3109 7422 3115 3113 3123 8600 10312 3119 1435 7043 8792 |
| hsa04310 | Wnt signaling pathway | 23291 387 6932 4041 1460 324 4772 5663 6934 7482 5515 4088 83439 54361 6424 8312 6500 7473 51384 1487 |
| hsa04940 | Type I diabetes mellitus | 3123 3458 3122 3113 2572 3117 3119 942 3109 3329 3115 |
| hsa04350 | TGF-beta signaling pathway | 4088 3458 5515 353500 387 3397 659 655 7043 6500 8200 657 7059 6667 |
| hsa05416 | Viral myocarditis | 3123 3122 3113 71 3117 3119 942 3109 1605 57644 3115 4629 |
| hsa05330 | Allograft rejection | 3123 3458 3122 3113 3117 3119 942 3109 3115 |
| hsa04916 | Melanogenesis | 805 83439 54361 163688 90993 112 808 6932 51384 7473 1638 3815 6934 7482 |
| hsa04510 | Focal adhesion | 56034 387 207 71 7148 1301 7059 7422 5501 7791 1101 7408 998 5728 3673 81 596 3691 3672 3381 |
| hsa05332 | Graft-versus-host disease | 3123 3458 3122 3113 3117 3119 942 3109 3115 |
| hsa05217 | Basal cell carcinoma | 83439 54361 8312 5727 324 6932 7473 51384 6934 7482 |
| hsa05150 | Staphylococcus aureus infection | 3123 3122 3113 4153 3117 3119 3426 3109 629 3115 |
| hsa04114 | Oocyte meiosis | 245711 5515 805 64682 23291 9232 163688 112 808 6790 6500 699 5501 9700 |
| hsa04340 | Hedgehog signaling pathway | 50846 353500 23291 54361 655 3549 5727 7473 51384 7482 |
| hsa05140 | Leishmaniasis | 3123 3458 3122 3113 7189 3117 3119 7043 3109 5970 3115 |
| hsa05412 | Arrhythmogenic right ventricular cardiomyopathy (ARVC) | 83439 786 71 3673 81 6932 784 3691 3672 1605 6934 |
| hsa04060 | Cytokine-cytokine receptor interaction | 3458 56034 659 6359 6368 4050 8200 7173 10663 7422 8600 1230 655 1435 5618 6358 7043 4982 657 3815 8792 2829 |
| hsa05210 | Colorectal cancer | 4088 6932 596 83439 324 387 207 8312 6934 7043 |
| hsa05320 | Autoimmune thyroid disease | 3123 3122 3113 3117 3119 942 3109 7173 3115 |
| hsa05310 | Asthma | 3123 3122 3113 3117 3119 3115 3109 |
| hsa04722 | Neurotrophin signaling pathway | 10019 805 7189 627 387 6272 207 998 163688 808 596 5663 5970 9252 |
| hsa04062 | Chemokine signaling pathway | 55970 387 1230 207 6359 6368 998 2793 6358 2788 112 2782 6777 10663 5970 2829 10235 |
| hsa05145 | Toxoplasmosis | 3123 10105 3458 3122 3113 7189 207 3117 3119 7043 3109 596 5970 3115 |
| hsa04672 | Intestinal immune network for IgA production | 3123 3122 3113 3117 3119 942 3109 3115 |
| hsa04520 | Adherens junction | 4088 83439 387 10580 998 71 81 1460 6932 6934 |
| hsa04270 | Vascular smooth muscle contraction | 805 387 800 3778 163688 135 112 808 10266 136 5501 4629 |
| hsa05410 | Hypertrophic cardiomyopathy (HCM) | 7134 786 71 3673 7043 5571 784 3672 3691 1605 |
| hsa04530 | Tight junction | 5515 387 207 998 71 5728 81 1460 23562 57644 84612 149461 4629 |
| hsa04145 | Phagosome | 3123 3122 3113 4153 9902 10312 71 3117 3119 3673 3109 5868 7059 3115 |
| hsa03040 | Spliceosome | 84991 3178 6631 4686 58517 8449 23451 23350 9343 6434 25804 9416 10291 |
| hsa05221 | Acute myeloid leukemia | 6932 83439 6776 6777 207 3815 5970 6934 |
| hsa04110 | Cell cycle | 4088 64682 993 9184 9232 7043 8379 6500 699 4171 4176 9700 |
| hsa04612 | Antigen processing and presentation | 3123 3458 3122 3113 3117 3119 8625 3109 3115 |
| hsa05414 | Dilated cardiomyopathy | 7134 786 71 3673 7043 112 784 3672 3691 1605 |
| hsa03013 | RNA transport | 253314 5976 4686 8669 8892 8890 23165 81929 8086 10460 100101267 79760 7341 |
| hsa04810 | Regulation of actin cytoskeleton | 2147 56034 85464 55970 387 71 324 26291 5501 5305 1073 998 3673 81 3672 3691 |
| hsa04514 | Cell adhesion molecules (CAMs) | 3123 3122 3113 8506 5788 3117 3119 942 3109 23562 3115 149461 |
| hsa04910 | Insulin signaling pathway | 805 253314 10580 207 163688 6720 2538 5571 808 3101 5501 5139 |
| hsa04512 | ECM-receptor interaction | 1101 3673 7148 7059 1301 3672 3691 1605 3381 |
| hsa05213 | Endometrial cancer | 6932 83439 324 207 5728 8312 6934 |
| hsa04144 | Endocytosis | 4088 7189 51160 387 998 11311 7043 9101 5868 382 93343 3815 2066 84612 84364 |
| hsa04010 | MAPK signaling pathway | 7189 627 55970 207 4763 26291 9252 10235 10746 786 998 4296 7043 784 5970 7786 6416 4137 |
| hsa05215 | Prostate cancer | 90993 6932 596 83439 56034 207 5728 5970 6934 |
| hsa05220 | Chronic myeloid leukemia | 4088 613 207 7043 6777 6776 5970 1487 |
| hsa04380 | Osteoclast differentiation | 3458 7189 8600 207 1435 2354 4982 7006 5970 4772 8792 |
| hsa03050 | Proteasome | 3458 5692 5719 7979 5686 23198 |
| hsa04070 | Phosphatidylinositol signaling system | 808 160851 805 8527 8525 5728 5305 163688 |
| hsa05322 | Systemic lupus erythematosus | 3123 3458 3122 3113 3117 3119 942 729 3109 81 3115 |
| hsa05016 | Huntington's disease | 548644 627 517 29796 4726 7417 23186 90993 4728 4701 83544 55967 6667 |
| hsa04914 | Progesterone-mediated oocyte maturation | 245711 64682 993 207 8379 112 699 5139 |
| hsa04150 | mTOR signaling pathway | 253260 253314 207 7422 3091 54541 |
| hsa04020 | Calcium signaling pathway | 5737 7134 805 7125 491 7417 163688 135 808 136 2066 1812 |
