# Supplementary material for: Multiple analyses of large-scale genome-wide association study highlight new risk pathways in lumbar spine bone mineral density
Source: Oncotarget. 2016 Apr 23;7(21):31429–39. doi: 10.18632/oncotarget.8948 (PMC5058768; doi:10.18632/oncotarget.8948)
Supplement: Supplementary file 5 [file oncotarget-07-31429-s005.doc]

Supplementary Table 4, The detailed genes in significant KEGG pathways using gene from PLINK

| Pathway ID | Pathway Name | Gene |
| --- | --- | --- |
| hsa05323 | Rheumatoid arthritis | 3123 3113 8600 10312 3117 3119 942 523 4050 7422 8792 3115 |
| hsa04060 | Cytokine-cytokine receptor interaction | 56034 6359 6368 91 4050 3449 8200 7173 10663 7422 8600 655 5618 6358 6366 4982 3815 8792 2829 |
| hsa04940 | Type I diabetes mellitus | 3123 3113 2572 3329 3117 3119 942 3115 |
| hsa05320 | Autoimmune thyroid disease | 3123 3113 3117 3119 942 7173 3449 3115 |
| hsa04010 | MAPK signaling pathway | 7189 627 4763 26291 9252 10235 2246 10746 3305 998 4790 2252 784 5970 7786 409 4137 |
| hsa04145 | Phagosome | 3123 3113 4153 10312 3117 3119 523 4360 3673 7059 4353 3115 |
| hsa04310 | Wnt signaling pathway | 4088 5515 387 6424 8312 324 1460 4041 51384 7473 4772 1487 |
| hsa04510 | Focal adhesion | 56034 1101 7791 387 7408 998 7448 3673 7148 5500 81 1301 7059 7422 |
| hsa05140 | Leishmaniasis | 3123 3113 7189 3117 3119 4790 5970 3115 |
| hsa05416 | Viral myocarditis | 3123 3113 3117 3119 942 6443 3115 1605 |
| hsa04810 | Regulation of actin cytoskeleton | 2246 2147 56034 85464 387 998 3673 2252 5500 81 324 26291 5305 1073 |
| hsa05330 | Allograft rejection | 3123 3113 3117 3119 942 3115 |
| hsa05332 | Graft-versus-host disease | 3123 3113 3117 3119 942 3115 |
| hsa05145 | Toxoplasmosis | 3123 10105 3305 3113 7189 3117 3119 4790 5970 3115 |
| hsa04672 | Intestinal immune network for IgA production | 3123 3113 3117 3119 942 3115 |
| hsa05310 | Asthma | 3123 3113 3117 3119 3115 |
| hsa00250 | Alanine, aspartate and glutamate metabolism | 790 84706 2673 2572 189 |
| hsa04062 | Chemokine signaling pathway | 387 6359 6368 998 4790 6358 6366 10663 409 5970 2829 10235 |
| hsa04142 | Lysosome | 9374 84572 10312 3074 2720 5476 9179 130340 3425 |
| hsa05150 | Staphylococcus aureus infection | 3123 3113 4153 3117 3119 3115 |
| hsa04612 | Antigen processing and presentation | 3123 3305 3113 3117 3119 3115 8625 |
| hsa04722 | Neurotrophin signaling pathway | 10019 805 7189 627 387 998 9252 5970 4790 |
| hsa04514 | Cell adhesion molecules (CAMs) | 3123 3113 8506 3117 3119 942 23562 3115 149461 |
| hsa04350 | TGF-beta signaling pathway | 4088 5515 387 3397 7059 8200 655 |
| hsa04512 | ECM-receptor interaction | 1101 1301 7059 7448 1605 3673 7148 |
| hsa03050 | Proteasome | 5692 5719 7979 5686 5718 |
| hsa04144 | Endocytosis | 4088 3305 7189 51160 387 998 409 3815 93343 84612 84364 |
| hsa04670 | Leukocyte transendothelial migration | 6494 387 7408 998 81 23562 7294 149461 |
| hsa05220 | Chronic myeloid leukemia | 4088 613 6776 5970 1487 4790 |
| hsa04520 | Adherens junction | 4088 81 1460 387 5770 998 |
| hsa04380 | Osteoclast differentiation | 7189 4982 8600 4772 5970 8792 4790 2354 |
| hsa04530 | Tight junction | 81 5515 1460 387 998 23562 84612 149461 |
| hsa05160 | Hepatitis C | 5515 7189 4790 3449 5970 23562 3659 149461 |
| hsa05217 | Basal cell carcinoma | 5727 324 51384 7473 8312 |
| hsa04340 | Hedgehog signaling pathway | 5727 50846 51384 7473 655 |
| hsa04114 | Oocyte meiosis | 245711 5500 5515 805 9700 9232 10393 |
| hsa03013 | RNA transport | 11097 8892 253314 5976 8890 10799 8086 79760 |
| hsa03010 | Ribosome | 6156 6234 6205 6164 6207 6204 |
